# γ-Secretase inhibition promotes cell death, Noxa upregulation, and sensitization to BH3 mimetic ABT-737 in human breast cancer cells

**DOI:** 10.1186/bcr3214

**Published:** 2012-06-15

**Authors:** Céline Séveno, Delphine Loussouarn, Sophie Bréchet, Mario Campone, Philippe Juin, Sophie Barillé-Nion

**Affiliations:** 1 Team 8 "Cell survival and tumor escape in breast cancer," UMR 892 INSERM/6299 CNRS/Université de Nantes, Institut de Recherche Thérapeutique de l'Université de Nantes, 8, quai Moncousu, BP 70721, 44007 Nantes Cedex 1, France; 2 Institut de Cancérologie de l'Ouest, Centre de Lutte contre le Cancer René Gauducheau, Boulevard Jacques Monod, 44805 Saint Herblain-Nantes Cedex, France; 3 Service d'Anatomie Pathologique, HGRL, CHU Nantes, Nantes, France

## Abstract

**Introduction:**

Inappropriate Notch signaling, downstream of γ-secretase activity, is understood to have tumor-promoting function and to be associated with poor outcome in cancer, of the breast in particular. The molecular basis of antitumoral effects of its inhibitors, however, remains poorly characterized. Moreover, the effects of their combination with the pro-apoptotic pharmacologic inhibitor of Bcl-2/Bcl-xL, ABT-737, have never been evaluated. In this study, we thus specifically addressed the biologic consequences of targeting γ-secretase and Bcl-2/Bcl-xL, alone or simultaneously, in breast cancer cell lines as well as in a novel human breast cancer *ex vivo *assay.

**Methods:**

By using *in vitro *2D or 3D cultures of breast cancer cells plus a novel preclinical short-term *ex vivo *assay that correctly maintains human mammary tissue integrity and preserves tumor microenvironment, we tested the effects of the pharmacologic γ-secretase inhibitor GSIXII used as a single agent or in combination with ABT-737.

**Results:**

We show herein that the γ-secretase inhibitor, GSIXII, efficiently induces apoptosis in breast cancer cell lines by a process that relies on the induction of Noxa, a pro-apoptotic Bcl2-homology 3 domain (BH3)-only protein of the Bcl-2 family that functions as an inhibitor of antiapoptotic Mcl1. GSIXII also targets mammary cancer stem-like cells because it dramatically prevents *in vitro *mammosphere formation. Moreover, combining GSIXII treatment with ABT-737, a BH3-mimetic inhibitor of additional antiapoptotic proteins, such as Bcl-2 and Bcl-xL, leads to both a synergistic apoptotic response in breast cancer cells and to an inhibitory effect on mammosphere formation. These effects are also found when a Notch transcriptional inhibitor, SAHM1, is used. Finally, we evaluated individual human tumor responses to γ-secretase inhibition alone or in combination with ABT-737 in *ex vivo *assays. Analysis of a series of 30 consecutive tumors indicated that a majority of tumors are sensitive to apoptosis induction by GSIXII and that association of GSIXII with ABT-737 leads to an enhanced induction of apoptosis in tumor cells.

**Conclusions:**

We thus provide evidence that γ-secretase, and downstream Notch signaling, are relevant targets in breast cancer. GSIXII, used as single agent or in combination with clinically relevant BH3-mimetics, is a promising innovative proapoptotic strategy to treat mammary tumors.

## Introduction

Notch signaling impinges on a wide variety of cellular processes, including cell-fate specification, cell proliferation, differentiation, apoptosis, and maintenance of stem cells. Deregulation of Notch signaling leads to several pathologic conditions, including cancer [1]. Notch was first identified as an oncogene in T-acute lymphoblastic leukemia with (7,9) chromosomal translocation [2] or activating mutation within *Notch1 *gene [3]. The Notch pathway also participates in oncogenesis through aberrant activation related to deregulated expression of Notch receptors or ligands, or the loss of a negative regulator, as described for Numb. Such inappropriate activation of the Notch pathway has been reported in many solid tumors, including breast cancer, in which it was linked to poor clinical outcomes [4-6]. Of note, the Notch pathway may have a direct oncogenic effect by its aberrant activation in cancer but may also be involved in feedback-reactivation process after conventional anticancer therapy, thus participating in chemoresistance. Indeed, this pathway is turned on in breast cancer cells, on tamoxifen treatment of estrogen receptor (ER)-positive tumors [7,8], or after HER2 inhibition in HER2-amplified tumors [9]. This is due to the capacity of estradiol or the HER2 pathway intrinsically to inhibit Notch activity. Another important point is that the mammary microenvironment can trigger Notch paracrine signaling to mammary cells, making a potent niche for mammary stem cells [10,11].

After ligand binding to Notch transmembrane receptors, a series of proteolytic reactions leads to the release of Notch intracellular domain (NICD), allowing its translocation into the nucleus, where it interacts with DNA-bound protein factor CSL (or CBF1) and recruits MAML family member coactivators, such as MAML1. These events lead to the formation of a trancriptional activator complex that drives the transcription of targeted genes [12].

The final proteolytic cleavage step mediated by the γ-secretase complex is critical for Notch-signaling activation, and its inhibition can be exploited through emerging pharmacologic drugs identified as γ-secretase inhibitors (GSIs). These new agents attenuate signaling from all four receptors and are being investigated as candidates in cancer therapy. Recent studies provided evidence that GSI treatment suppressed growth of breast cancer cells, increasing the interest in validating this novel therapeutic approach [13-16].

A better understanding of molecular mechanisms involved in the antitumoral effect of Notch inhibition is needed to develop a comprehensive use of Notch inhibitors such as GSI. γ-Secretase activity and Notch signaling appear to be critical for cell survival [17,18], but evaluating how exactly their inhibition affects survival pathways in cancer cells remains to be performed. Along this line, it must be noted that the effects of γ-secretase inhibition have not been systematically assessed. In particular, their effects on intact human tumors in the presence of their microenvironment have not been evaluated. Aberrant survival signaling is a frequent feature of cancer cells, in part due to the acquisition of an increased apoptotic threshold leading to tumor chemoresistance [19]. This process often arises from the deregulation of Bcl-2 family members. This family is divided into three categories, (a) the antiapoptotic proteins (Bcl-2, Bcl-xL, and Mcl-1); (b) the proapoptotic BH3-only proteins, such as Noxa, Puma, and Bim; and (c) the proapoptotic multidomain proteins (Bax, Bak) that function downstream of the former. This family of proteins maintains a subtle survival/cell-death balance by regulating mitochondrial integrity, caspase activation, and consequent cell demolition. Antiapoptotic proteins promote survival, in great part, by physically interacting with the BH3 domain of their proapoptotic counterparts via a well-characterized binding interface. Subtle yet significant differences exist in the BH3-binding interface of each Bcl-2 homologue, so that promiscuous but also selective interactions occur between these proteins and multidomain or BH3-only proteins. For instance, Bim or Puma interacts with all known Bcl-2 homologues, whereas Bad interacts preferentially with Bcl-2 and Bcl-xL, and Noxa, with Mcl-1 [20]. Thus, Bcl-2 homologues exert complementary effects on cell survival, and their simultaneous inhibition is expected to promote efficient cancer cell death.

The pivotal role of the Bcl-2 family in the apoptotic pathway has stimulated considerable interest in developing anticancer agents that specifically act to restore apoptotic cell death [21]. The BH3 mimetic, ABT-737, is a promising compound that potently binds to and neutralizes the prosurvival proteins Bcl-2, Bcl-xL, and Bcl-w, but not Mcl-1 or A1 [22]. Thus, expression of Mcl-1 confers resistance to ABT-737 when used as single agent and, conversely, approaches that lead to downregulation or inhibition of Mcl-1 are expected to enhance sensitivity to this compound.

Based on these premises, we investigated the impact of Notch inhibition on the apoptotic threshold in breast cancer cells, by focusing our analysis on the Bcl-2 family of proteins. We first pointed out that the γ-secretase inhibitor GSIXII, used as single agent, triggers apoptosis *in vitro *in breast cancer cells. It also exerts an inhibitory effect on breast cancer cells that have a stem-like phenotype, as does the Notch transcriptional inhibitor SAHM1. Importantly, GSIXII treatment also induced an apoptotic response in numerous intact breast tumors tested in an *ex vivo *assay developed in our laboratory. We further demonstrated that the GSIXII apoptotic effect depended mainly on the induction of Noxa, a BH3-only protein that inhibits Mcl-1. Consistently, GSIXII treatment combined with Bcl-2/Bcl-xL inhibition by ABT-737 potently enhanced the proapoptotic response of the breast cancer cells, including in *ex vivo *specimens. Thus, our results highlight the clinical relevance of targeting γ-secretase and downstream Notch signaling in breast cancer, especially in combination with the Bcl-2/Bcl-xL inhibitor ABT-737.

## Materials and methods

### Reagents and cell lines

MCF7, BT549, MDAMB231, ZR75.1, and T47D cell lines were from American Type Culture Collection, and Cal51, from DSMZ (Braunschweig, Germany). All cell lines were cultured by following the suppliers' recommendations.

SAHM1 and γ-secretase inhibitor XII (GSIXII, z-Ile-Leu-CHO) were purchased from Calbiochem (Darmstadt, Germany, Bortezomib and ABT-737 from Selleck Chemicals (Houston, TX, USA), and the pan-caspase inhibitor QVD-OPH, from R&D Systems (Abingdon, UK). Antibodies against cleaved Notch1 (N1ICD) were purchased from Cell Signaling (Saint Quentin en Yvelines, France); Puma, from Epitomics (Nanterre, France); and Bim and Noxa, from AbCam (Cambridge, UK). Antibodies to Bax and Actin were from Dako (Trappes, France) and Millipore, (Molsheim, France) respectively. Antibody against cleaved (that is, activated) caspase-3 used in IHC was from BD Bioscience (le Pont de Claix, France).

### Apoptosis assays

Cell death was assessed with Apo2.7 (Beckman Coulter, Villepinte, France) staining and confirmed by an Annexin-V binding assay (Beckman Coulter, Villepinte, France), performed according to manufacturer's instructions. Flow-cytometry analysis was performed on a FACSCalibur by using the CellQuestPro software.

### Mammosphere-formation assay

MCF7 or BT549 cells treated with the indicated treatment or siRNA were plated as single cells in ultra-low-attachment plates (Corning, Avon, France) at low density (500 viable cells/cm^2^). They were grown in serum-free mammary epithelial cell growth medium containing DMEM-F12 (Sigma, Saint-Quentin Fallavier, France) supplemented with B27 (Gibco, Saint Aubin France) and MEGM singlequots (Lonza, Levallois-Perret, France), as previously described [23]. Mammosphere-forming units (MFUs) were counted as the number of mammospheres ≥ 50 μm. Mammosphere formation of the second and third generations was investigated in the presence of GSIXIII, or not, after trypsin treatment of the first- and second-generation mammospheres, respectively.

### Immunoblot analysis

Patient samples for immunoblots were snap-frozen into liquid nitrogen and then prepared according to [24]. Cell-lines samples were prepared as previously described [25]. Fifty micrograms of protein was loaded for each lane and separated by 10% or 12.5% SDS-PAGE, then electrotransfered to PVDF membranes. Western blot analysis was performed by standard techniques with ECL detection (Pierce, IllKirch, France).

### RNA interference

Cells were transfected by using Lipofectamine RNAiMax 2000 (Invitrogen), according to the manufacturer's instructions. Medium was changed 6 hours later, and compounds were added after 24 hours. The following siRNAs were used: control siRNA (sc44230) from Santa Cruz, siRNA Bim (6461) from Cell Signaling, siRNA Puma from Dharmacon (Lafayette, USA), siRNA Noxa (AC2Z4U4) from Ambion (Saint Aubin, France), and siRNA Bax from IDT (Leuven, Belgium).

### Quantitative PCR

Total RNA was isolated from cell lines with RNeasy Plus mini kit (Qiagen, France). The quality of the RNAs was assessed by analysis of the 28S:18S rRNA ratio by using the RNA 6000 Nano Assay kit and the Agilent 2100 bioanalyzer (Agilent Biotechnologies). Then 500 ng of total RNA was reverse transcribed by using the superscript III reverse transcriptase and random hexamers (Life Technologies, Saint Aubin, France). Quantitative PCR was done by using the Maxima SYBR Green/ROX qPCR Master Mix (Life Technologies) and the MX4000 instrument (Stratagene, Basel, Switzerland), according to the manufacturer's instructions. To control the specificity of the amplified product, a melting-curve analysis was done. No amplification of unspecific product was observed. Primer sequences were 5'-GCTGGAAGTCGAGTGTGCTA-3' (forward) and 5'-CCTGAGCAGAAGAGTTTGGA-3' (reverse) for *Noxa*. *RPLPO *AACCCAGCTCTGGAGAAACT (forward) and CCCCTGGAGATTTTAGTGGT (reverse), *HPRT1 *5'-ATGCTGAGGATTTGGAAAGG-3' (forward) and 5'-GATGTAATCCAGCAGGTCAGC-3' (reverse) and *RSP18 5*'-ATCCCTAAAAGTTCCAG-3' (forward) and 5'-CCCTCTTGGTGAGGTCAA-3' (reverse) were used for normalization. Relative quantification was carried out by using the ΔΔCt method.

### Promoter-reporter activity assay

The ability of NICD to bind to CBF1 and activate gene transcription was measured by the transfection of luciferase reporter plasmids that contain four copies of a binding site for CBF1 (CBF1-Luc) or mutated CBF1 (mCBF1-Luc) that were a kind gift from Dr. Diane Hayward (Johns Hopkins University, Baltimore, MD, USA) [26]. Cells were transfected by using Lipofectamine 2000 (Invitrogen). Medium was changed 6 hours later, and treatment was added after 24 hours. Cells were harvested 48 hours after transfection and analyzed by using the Stop&Glow kit (Promega, Lyon, France) and following the manufacturer's instructions. Results were expressed as ratios between the CBF1-Luc-transfected samples and the mCBF1-Luc-transfected one for each cell line in each condition, in three independent experiments.

### Lentiviral infection

Recombinant lentivectors were produced by transient transfection of the transducing vector into 293T cells with two packaging vectors: pMD.G, a plasmid expressing the VSV-G envelope gene (Addgene plasmid 12259, Addgene, Cambridge, MA), and pCMVDeltaR8.91, a plasmid expressing the HIV-1 gag/pol, tat, and rev genes (Addgene plasmid 8455) associated with a GFP control plasmid (Addgene plasmid 17618) or plasmid coding for N1ICD and GFP with two independent internal promoters (Addgene plasmid 17626), as described previously [27]. Cells were infected for 24 hours before treatment with GSIXII for 48 hours, and apoptosis was assessed on GFP^+ ^cells by using Apo2.7 staining followed by flow-cytometry analysis.

### Preclinical breast cancer *ex vivo *assay

Fresh human mammary samples were obtained from patients with invasive carcinoma after surgical resection at the Institut de Cancérologie de l'Ouest, René Gauducheau, Nantes, France. As required by the French Committee for the Protection of Human Subjects, informed consent was obtained from study patients to use their surgical specimens and clinicopathologic data for research purposes, and the local ethics committee approved protocols.

The tumors were cut into thin slices (250 μm) by using a vibratome (Microm International, ThermoFischer Scientific, Illikirch, France) and incubated for 48 hours with or without 15 μ*M *GSIXII. Slices were then fixed in 10% buffered formalin and were paraffin embedded. Sections (3 μm) were then cut for standard histologic analysis assessed by hematoxylin-eosin-saffron (HES) coloration. Immunohistochemistry (IHC) was performed to assess tumoral cell apoptosis with cleaved caspase-3 antibody. In brief, after deparaffinization in xylene and rehydration, endogenous peroxidase activity was blocked with 3% hydrogen peroxide. Samples were steamed for antigen retrieval with citrate buffer (pH 6.0). Slides were incubated for active caspase-3 (clone C92-605, dilution 1:1,200) on an automated immunostainer (Autostainer Plus, Dako) by using a standard labeled streptavidin-biotin method (Dako, LSAB+, Dako REAL Detection Systems kit) followed by 3,3'-diaminobenzidine chromogen detection. Immunostained slides were counterstained with hematoxylin (Dako, Trappes, France). Negative controls (omission of the primary antibody) were included in each run. Active caspase-3 immunostained cells were assessed according the percentage of labeled cells in 200 carcinomatous cells counted. Nonneoplastic cells were excluded from counting.

### Statistical analysis

Statistical analysis was performed by using a one-tailed paired Student *t *test and one-way ANOVA test on GraphPad Prism. Errors bars represent standard errors of the mean (SEM). The symbols correspond to a *P *value inferior to *0.05, **0.01, or ***0.001, and ns for not statistically significant.

## Results

### Notch inhibition induced growth arrest and cell death in breast cancer cells

**Figure 1:**
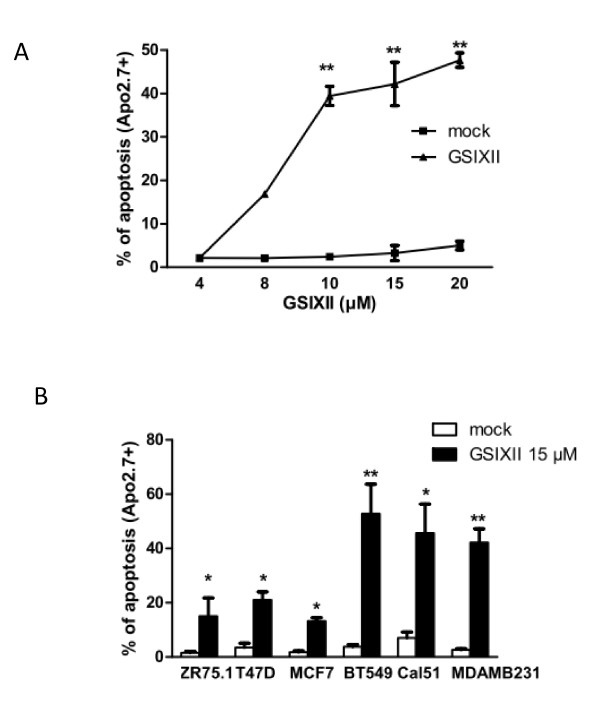
**GSIXII induced apoptosis in ER^+ ^or ER-**/**HER2^- ^breast cancer cell lines**. **(A) **MDAMB231 cells were treated with increasing GSIXII doses and analyzed with flow cytometry after Apo2.7 immunostaining. Data represent (percentage of Apo2.7-positive cells) and the means ± standard errors (SEM) of three independent experiments. **(B) **Three ER^+ ^human breast cancer cell lines (ZR75.1, T47D, and MCF7) and three ER-/HER2- (BT549, Cal51, and MDAMB231) were treated with GSIXII, 15 μ*M*, for 48 hours and then analyzed with flow cytometry after Apo2.7 immunostaining. Represented data are the means ± SEM of three independent experiments.

To investigate the proapoptotic effects of the γ-secretase inhibitor GSIXII and to define the range of active concentrations, we treated the breast cancer cell line MDAMB231 with increasing concentrations (4 to 20 μ*M*) for 48 hours before evaluation of apoptosis by measuring the expression of Apo2.7 antigen (whose expression is restricted to dying cells) by using flow-cytometry analysis. In comparison to DMSO (mock) treatment, GSIXII treatment induced specific apoptosis, and concentrations from 8 μ*M *to 15 μ*M *triggered increasing cell death (Figure 1A). The concentration of 15 μ*M*, corresponding to a plateau (inducing 40% of apoptotic cells), was used. We further tested a panel of six human breast cancer cells lines either expressing estrogen receptor (ER^+^) (ZR75.1, T47D and MCF7) or not (ER^-^, without amplification of the *HER2 *oncogene) (BT549, Cal51, MDAMB231), for their cell-death response to this treatment (Figure 1B). All of them showed significant sensitivity to GSIXII. Interestingly, ER^-^/HER2^- ^cell lines exhibited higher sensitivity to GSIXII (40% versus 20% in ER^+ ^cell lines), as previously observed by Lee *et al*. [15]. Apoptotic response to GSIXII treatment was further confirmed by Annexin-V binding assay, as shown for BT549, MDAMB231, and MCF7 cell lines in Additional file 1.

Numerous observations confirmed that GSIXII potently triggered an apoptotic response in breast cancer cells through inhibition of Notch activity in the breast cancer cells used.

**Figure 2:**
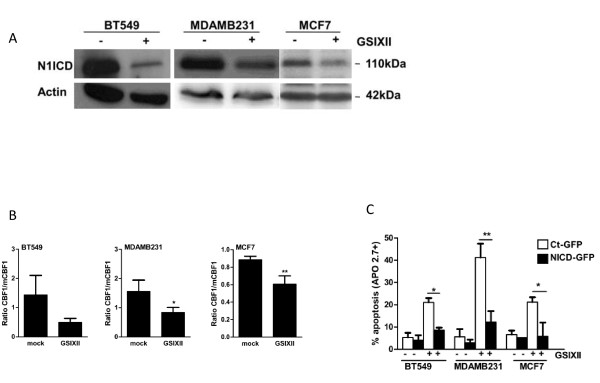
**GSIXII inhibited the Notch signaling pathway in breast cancer cell lines**. **(A) **Breast cancer cell lines were evaluated for Notch1-ICD expression with immunoblot analysis after treatment by GSIXII 15 μ*M *(+) or mock (-) for 48 hours, by using actin as loading control. **(B) **Notch activity was evaluated with CBF1- or mutated CBF1-luciferase promoter assay after treatment by GSIXII, 15 μ*M*, for 24 hours, compared with mock-treated cells. Represented data are means of CBF1/mutated CBF1 ratios ± SEM of three independent experiments. **(C) **Cells were infected with control-GFP (white) or N1ICD-GFP (black) lentivectors and treated with 15 μ*M *GSIXII for 48 hours. Apoptosis of GFP-positive cells was assessed with Apo 2.7 staining followed by flow-cytometry analysis. Represented data are means ± SEM of three independent experiments.

First, we evaluated, with immunoblot analysis, the expression of the active form of Notch1, N1ICD, in GSIXII-treated cells compared with control cells, and found that GSIXII treatment downregulated N1ICD expression (Figure 2A).

Second, we measured Notch transcriptional activity, with a Notch promoter luciferase assay containing CBF1 or mutated CBF1 boxes, and this assay pointed out the efficient inhibition of Notch-driven luciferase transcription on GSIXII treatment (Figure 2B).

Third, overexpression of the human N1ICD obtained by lentiviral infection efficiently protected breast cancer cells from GSIXIII-induced apoptosis (Figure 2C). Altogether, these results indicate that GSIXII potently interfered with Notch activity, and that this effect contributed in its impact on cell survival.

γ-Secretase inhibitors may also inhibit proteasome activity, and this effect might contribute to their biologic activity. We thus compared the effects of GSIXII and the well-known proteasome inhibitor bortezomib on both proteasome activity and cell survival. These assays showed that GSIXII had a significant effect on proteasome activity (see Additional file 2A). However, bortezomib treatment that recapitulated this effect did not promote cell death, in contrast to treatment with GSXII (see Additional file 2B). The lack of correlation between inhibition of proteasome activity and apoptotic activity in these assays indicates that apoptosis induction by GSIXII cannot solely rely on its ability to inhibit proteasome activity, even though we cannot formally rule out that this effect contributes to cell-death induction.

### GSI treatment triggered Noxa-dependent apoptosis in breast cancer cells

**Figure 3:**
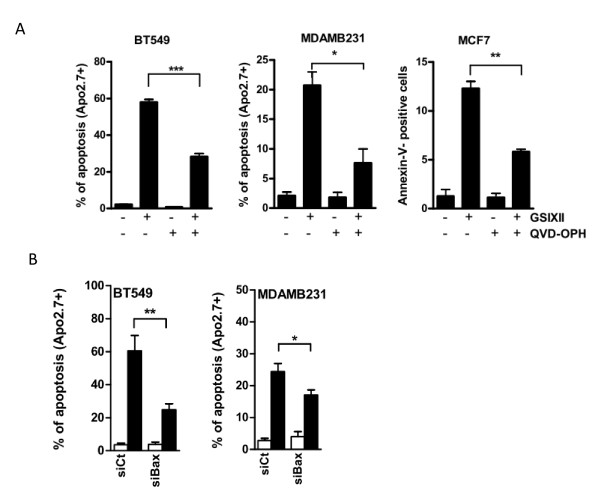
**GSIXII-induced cell death involved a canonic intrinsic apoptotic pathway**. Cell death triggered by GSIXII depended on caspase activity. Breast cancer cells were pretreated with the pan-caspase inhibitor QVD-OPH (20 μ*M*) before GSIXII treatment at 15 μ*M *for 48 hours and analyzed for Apo2.7 or Annexin-V staining (more suitable for the MCF7 caspase-3 deficient cell line). Data are the means of positive cells ± SEM; *n *= 3. **(B) **Bax siRNA protected breast cancer cells from GSIXII-induced apoptosis. SiRNA (control (Ct) or Bax)-transfected cells were treated with 15 μ*M *GSIXII before Apo2.7 immunostaining and flow-cytometry analysis. Represented data are the means of positive cells ± SEM, from three independent experiments.

The proapoptotic effects of GSIXII were strongly prevented by co-treatment with the chemical pancaspase-inhibitor QVD-OPH in all breast cancer cell lines (as shown for three of them in Figure 3A). As Bax is a major actor in the onset of apoptosis by the mitochondrial pathway, the impact of its knockdown by RNA interference on GSIXII induction of cell death was evaluated. Results shown in Figure 3B indicate that siRNA targeting Bax significantly preserved breast cancer cells from the deleterious effects of GSIXII. Thus, GSIXII-induced apoptosis appears to occur mainly through the canonic mitochondria-dependent pathway requiring Bax and caspase activation.

**Figure 4:**
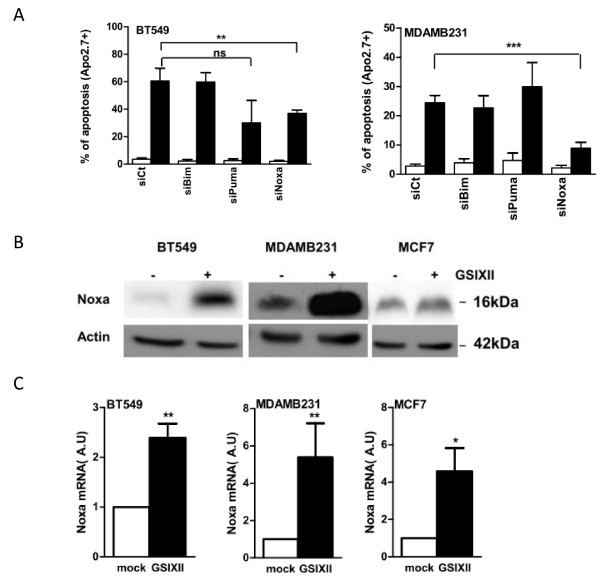
**Noxa induction triggered GSIXII-induced apoptosis**. **(A) **Breast cancer cells were first transfected by siRNA targeting Bim, Puma, or Noxa, or control siRNA (siCt), and then treated or not with GSIXII for 48 hours and analyzed with flow cytometry after Apo2.7 immunostaining. Represented data are the means of positive cells ± SEM, from three independent experiments. SiRNA effects were compared with corresponding controls. **(B) **Expression of Noxa protein was assessed with immunoblot after 48 hours of treatment of GSIXII, 15 μ*M*, in indicated breast cancer cell lines. **(C) **Noxa mRNA is induced by a GSIXII treatment. Quantitative PCR was performed on cell lines after 48 hours of treatment with mock (white) or GSIXII (black) and quantified as arbitrary units (au) compared with the mock-treated condition. Represented data are the means of positive cells ± SEM, from three independent experiments.

To investigate further the molecular pathways involved in GSIXII induction of cell death, we performed siRNA-based experiments against Noxa, Bim, or Puma before treating cells with GSIXII. Of major importance, the sole depletion of Noxa by RNA interference led to decreased cell sensitivity to GSIXII in all cell lines tested (Figure 4A and Additional file 3A). In contrast, neither Puma nor Bim depletion had a significant impact on the cell-death response to GSIXII. Of note, protection against cell death by Noxa knockdown was not complete, but this might rely on residual partial Noxa expression after Noxa siRNA treatment (see Additional file 3B). Thus GSIXII induces cell death preferentially by a Noxa-dependent cell-death pathway.

We then assessed the expression of the BH3-only proapoptotic proteins, Bim, Puma, and Noxa, with immunoblot analysis on treatment with GSIXII. In all breast cancer cell lines, a strong induction of Noxa protein expression was evidenced in response to GSIXII treatment (Figure 4B). In contrast, Puma or Bim expression was not enhanced (as shown in MDAMB231 in Additional file 4). The better to understand the mechanisms involved in Noxa protein accumulation on GSIXII treatment, RTqPCR analysis was performed to quantify Noxa mRNA. Data indicated that GSIXII induced Noxa mRNA, arguing for regulation of Noxa expression at a transcriptional level (Figure 4C).

### GSIXII treatment strongly impaired *in vitro *mammosphere formation

Transformed mammary epithelial cells, including established breast cancer cell lines, exhibit an inherent phenotypic plasticity and harbor a subpopulation of cancer-initiating cells with features resembling these of stem cells. The latter cells, which are characterized by numerous criteria, including their ability to form spherical colonies in nonadherent fetal bovine serum-free culture conditions (mammospheres), were frequently described as being resistant to cell-death induction by numerous stimuli, and they may therefore rely on survival signals distinct from the bulk population. Moreover, the Notch pathway might be involved in cell stemness. We thus evaluated whether GSIXII treatment had an impact on mammosphere formation by breast cancer cell lines and whether this relied on cell-death induction.

**Figure 5:**
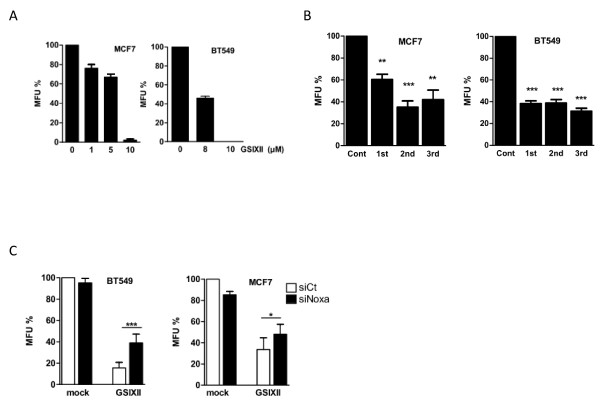
**Notch inhibition decreased mammosphere formation**. **(A) **MCF7 and BT549 cell lines were evaluated for their mammosphere-formation capacity with GSIXII treatment with indicated concentrations. Represented data are the means of MFU% compared with the mock-treated condition ± SEM, from three independent experiments. **(B) **The capacity of MCF7 and BT549 cells to form mammospheres at the first (1st), second (2nd), and third (3rd) generations was assessed in the presence or not of 8 μ*M *GSIXII added at the beginning of the MFU assay. Represented data are from three independent experiments. Statistical analysis compared mammosphere formation in each serial generation with its mock-treated control. **(C) **SiRNA targeting Noxa (black bar) significantly rescued mammosphere formation in BT549 and MCF7 cells on GSIXII or mock treatment compared with siRNA control (white bar).

**Figure 6:**
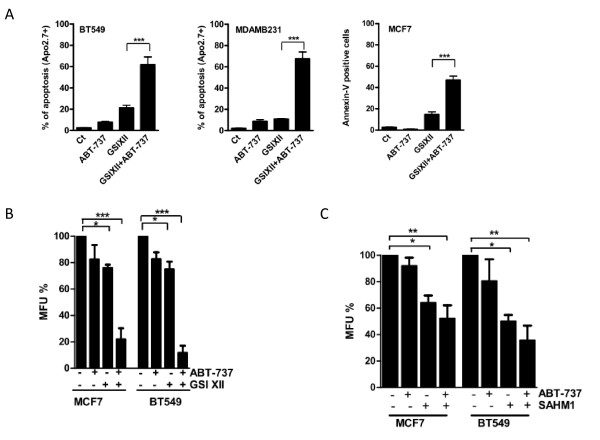
**GSIXII synergized with ABT-737 to trigger apoptosis in breast cancer cells**. Breast cancer cell lines were incubated for 48 hours with 10 μ*M *GSIXII or DMSO (Ct) in combination or not with ABT-737, 1 μ*M*. Then apoptosis was evaluated with Apo2.7 or Annexin-V staining and flow-cytometry analysis. Represented data are the means of positive cells ± SEM, from three independent experiments. **(A) **Suboptimal concentrations of GSIXII (5 μ*M*) and 1 μ*M *ABT-737 were used alone or in combination in MFU assay in MCF7 and BT549 cell lines. Results were obtained from three independent experiments and compared with mock-treated condition. **(B) **The 20 μ*M *SAHM1 was used alone or in combination in MFU assay in MCF7 and BT549 cell lines. Results were obtained from three independent experiments and compared with the mock-treated condition.

A dramatic decrease in mammosphere formation was observed after GSIXII treatment of MCF7 or BT549 cell lines compared with mock-treated cells (Figure 5A). This effect was recapitulated by the SAHM1 cell-permeable peptide (a dominant-negative fragment of MAML1 that specifically prevents assembly of the active transcriptional complex and blocks Notch transcription activity [28]), used at 20 μ*M *(Figure 6C). In addition, GSIXII not only inhibited first-generation mammosphere formation but also decreased the mammosphere formation of second and third generations (Figure 5B), which are further enriched in self-renewing cells. This argues that the treatment affects not only cells that can give progeny, but also cells that can self-renew. Of importance, Noxa depletion by RNA interference combined with GSIXII treatment partially but significantly rescued mammosphere formation (Figure 5C). Thus, GSIXII potently prevents mammosphere formation, and this effect relies, at least in part, on Noxa-dependent cell-death mechanisms. This argues for the capacity of GSIXII to target mammary stem-like cells.

### GSIXII and the BH3 mimetic ABT-737 strongly synergized to induce apoptosis in breast cancer cells

As GSIXII induced the expression of proapoptotic Noxa, which inhibits the survival activity of Mcl-1, we inferred that its combination with the BH3 mimetic ABT-737, which targets Bcl-2 and Bcl-xL but not Mcl-1, might improve apoptosis induction in breast cancer cells. We observed that combined treatment of breast cancer cell lines with a suboptimal concentration (that is, 8 μ*M *GSIXII and 1 μ*M *ABT-737) strongly synergized to induce cell death (Figure 6A). In these conditions, GSIXII induced cell-death rates lower than 20%, and ABT-737 induced death rates lower than 10%, whereas the combination of both drugs triggered cell-death rates ranging from 50% to 70%. Interestingly, this synergy was also observed when using the other γ-secretase inhibitor DAPT in combination with ABT-737 (see Additional file 5).

To confirm that Noxa induced on treatment with GSIXII functions as an inhibitor of Mcl-1, we further evaluated its interaction with Mcl-1 on GSIXIII treatment, with co-immunoprecipitation assays. We observed an increase of the interaction in the GSIXII-treated cells compared with mock-treated cells (see Additional file 6), demonstrating that Noxa could sequester Mcl-1 in treated cells. In addition, the presence of Noxa was greatly decreased in the Mcl-1-immunodepleted supernatants of GSIXII-treated cell lysates compared with the corresponding not-depleted ones, indicating that GSIXII-induced Noxa was in the majority complexed to Mcl-1 (data not shown). Interestingly, the GSIXII and ABT-737 combination led to inhibition of mammosphere formation in both MCF7 and BT549 cell lines (Figure 6B). The SAHM1 and ABT-737 combination also decreased mammosphere formation (Figure 6C). These results argue that potent apoptotic synergy is induced by γ-secretase inhibitors and ABT-737 in stem-like breast cancer cells, as well as in more-differentiated cells.

### Preclinical evaluation of GSI treatment on human mammary tumors

The tumor microenvironment is particularly important for Notch activation. We thus developed a model of 3D culture of human primary breast tumors in which the architectural integrity of the tumor, including its microenvironment, is preserved. In brief, fresh tumors were rapidly cut into thin slices and incubated in full medium alone or with drugs (GSIXII+/- ABT-737) for 48 hours. Tumor slices were then paraffin embedded and analyzed with IHC for active caspase-3 expression, as a marker of apoptotic response. We studied a series of 30 consecutive primary tumors from patients with untreated breast cancer for their sensitivity to the Notch inhibitor GSIXII with this short-term *ex vivo *culture of human breast cancer tissues.

**Figure 7:**
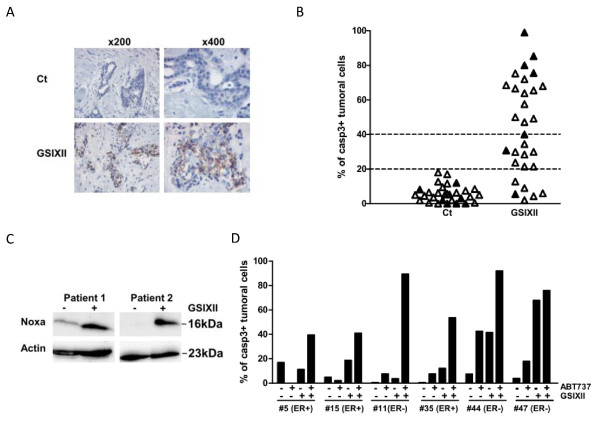
**GSIXII induced apoptosis in primary breast tumor cells in *ex vivo *assay**. Active caspase-3 immunostaining analyzed with immunohistochemistry in one of the human breast tumors 48 hours after 15 μ*M *GSIXII treatment or untreated (Ct). Magnification, ×200 and ×400. **(A) **23 ER^+ ^(Δ) or 7 ER^- ^(▲) primary human tumor samples were cultured 48 hours with 15 μ*M *GSIXII or not treated (Ct), as described in Materials and methods, and then analyzed for active caspase-3 with immunohistochemistry. Data are represented as percentage of tumoral cells positive for active caspase-3 immunostaining in each specimen. **(B) **Noxa expression was induced in breast tumor samples after 48-hour GSIXII treatment compared with the untreated condition, as shown in two samples assessed with immunoblot analysis. **(C) **The GSIXII and ABT-737 combination enhanced apoptosis triggering in breast cancer tumors. Six human primary tumor samples (three ER^+ ^and three ER^-^) were cultured for 48 hours with or without 15 μ*M *GSIXII in combination with 1 μ*M *ABT 737 or not for 48 hours, in *ex vivo *assay. The percentage of active caspase-3-positive tumoral cells was then established with immunohistochemistry. A one-way ANOVA test performed on the presented cohort of tumors indicates that the combination (GSIXII+ABT-737) was significantly better than either GSIXII or ABT-737 single treatment (** and ***, respectively).

To evaluate the specific response to GSIXII of each tumor sample, we systematically kept one slice untreated (to evaluate spontaneous rates of cell death) and treated another slice from the same tumor with 15 μ*M *GSIXII for 48 hours. Apoptosis was evaluated by counting the percentage of tumor cells that stained positive with an anti-active caspase-3 antibody, as evaluated by IHC analysis of the specimen fixed after incubation in either condition (Figure 7A); 23 ER-positive and seven ER-negative tumors were included in this preclinical study.

Investigation of untreated slices showed low cell-death rates in each tumor, with the mean percentage of active caspase-3-positive cells in these specimens reaching 5.7%. In comparison, the mean percentage of active caspase-3-positive cells in GSIXII-treated specimens was 44%. To classify individual tumors according to their apoptotic response to GSIXII, we arbitrarily defined a positive threshold above 17% of active caspase-3-positive tumor cells (corresponding to the highest score in untreated samples). Of 30 specimens, 24 showed a response to GSIXII above this threshold and can thus be considered GSIXII-sensitive tumors (Figure 7B). In contrast, six of 30 GSIXII-treated specimens showed cell-death rates that were undistinguishable from those found in control untreated specimens, which defined them as GSIXII-resistant tumors. Among sensitive specimens, we could identify two groups: an intermediate group of nine tumors that displayed 17% to 40% positive cells and a highly GSIXII-sensitive group of 15 tumors showing more than 40% of apoptotic cells under the conditions used. Importantly, a robust correlation was noted between the percentage of active caspase-3 tumor cells and tumor cell integrity, as evaluated with the standard hematoxylin-eosin-saffron staining performed on the same sample (see Additional file 7). This strongly suggests that the effects of GSIXII treatment on the tumor samples in this *ex vivo *test predominantly rely on an apoptotic response, which can be marked and quantified by caspase-3 activation. In addition, and consistent with this, Noxa induction could be detected in breast cancer tissues after GSIXII *ex vivo *treatment, as shown in two sensitive tumors (for which we obtained sufficient material to perform immunoblot analysis of untreated and GSIXII-treated specimens) compared with the corresponding untreated tissues (Figure 7C).

To evaluate whether ABT-737 treatment might enhance the apoptotic response of breast tumor samples to GSIXII induction of cell death, we also regularly treated, from the same series of tumor samples, one additional slice with 1 μ*M *ABT-737 and another one with a combination of GSIXII and ABT-737 before evaluation of the apoptotic response, as described earlier. Six specimens (three ER^+ ^and three ER^-^) proved to be informative in these assays, in that their apoptotic response to GSIXII and ABT-737, used as single agents, gave sufficiently low apoptotic responses, thus allowing synergy detection. Three of these specimens were GSXII resistant, one intermediate and two GSIXII-sensitive tumors. Moreover, regarding the ABT-737 response, four specimens were resistant, one was intermediate, and one, mildly sensitive. In all cases, the combination of ABT-737 treatment with that of GSIXII led to significantly enhanced cell death compared with that induced by each compound alone (Figure 7D). We conclude that at least some additivity occurs in the effects of the two compounds in both GSIXII-sensitive samples 44 and 47 and significant synergy in the four remaining tumors, for which the response to the combined treatment is higher that the sum of those obtained for each of the treatment-alone tumors.

## Discussion

Aberrant activation of the Notch pathway has been involved in solid-tumor pathogenesis, triggering protection against apoptosis or increased cell proliferation, yet the molecular basis for these effects remains unclear. To investigate these, and because the γ-secretase complex is a critical step in Notch-pathway activation, we evaluated the cell-death effects of inhibition of γ-secretase activity by GSIXII in breast cancer. Our results clearly indicate that GSIXII elicited potent apoptosis in breast cancer cells and that this effect occurred through the strong induction of the proapoptotic BH3-only protein Noxa. Of note, we showed that GSIXII treatment truly inactivated the Notch pathway, because it decreased both the expression of the Notch1 active form (N1ICD) and the global Notch transcriptional activity. Importantly, N1ICD overexpression rescued breast cancer cells from GSI-induced apoptosis. These latter results strongly argue that the potent cell-death effect of GSIXII relies on the inhibition of Notch processing into its active form. We cannot formally rule out, however, that additional effects, such as that on proteasome activity previously reported for the structurally related GSI-I [29], contribute to cell-death induction, but preliminary data with DAPT, that was described as a specific γ-secretase inhibitor devoid of inhibitory effect on proteasome activity by Han and colleagues [29], could also sensitize breast cancer cells to cell death. Importantly, the cytotoxic effects of GSIXII could be detected not only in the bulk of breast cancer cell lines but also in their stem cell-like compartment. This effect most likely results from Notch inhibition, as it was recapitulated by the Notch transcription factor inhibitor SAHM1. Intriguingly, this compound was more effective, in our hands, on this subpopulation enriched in self-renewing cells than on the bulk of cell lines. This may be due to a weak ability of the peptide to enter cells and/or to a higher dependency on Notch signaling of the stem-like cells compartment compared with the bulk population. Such a specific effect of Notch inhibition on mammosphere cultures has been observed by Farnie and colleagues [30,31]. Importantly, freshly explanted human breast cancer cells maintained in their microenvironment are also sensitive to induction of apoptosis by GSIXII. In addition, simultaneous treatment with GSIXII and the Bcl-2/Bcl-xL inhibitor ABT-737 led to synergistic effects in all three paradigms.

Altogether, our results strongly argue for the use of the γ-secretase inhibitors in breast cancer therapy, especially in combination with Bcl-2/Bcl-xL inhibition, which may help to reduce the dose of GSI used.

In an attempt to define molecular mechanisms involved in apoptosis triggered by GSIXII treatment, we first demonstrated that it was related to Bax and the caspase-dependent pathway. We then identified the BH3-only Bcl-2 family member Noxa as a pivotal actor. Indeed, its expression was strongly induced on GSIXII treatment, in breast cancer cell lines as well as in human primary breast tumors. Moreover, its knockdown by RNA interference potently blocked apoptosis in breast cancer cell lines, as previously observed in melanoma cell lines with GSI-I treatment [32]. We also detected Noxa mRNA accumulation on GSIXII treatment, arguing for an increase of its gene transcription rather than stabilization of the protein.

Previous studies have indicated Bcl-2 family members as major regulators of apoptosis triggering by Notch inhibition. Some reported the decrease of antiapoptotic Bcl-2 members, such as Bcl-xL, on GSI treatment in cancer cells [14,16]. Others described inefficient p53 response after treatment by potent p53 activators, such as genotoxic drugs acquired on Notch activation [17]. Of note, activated Notch1 could suppress Noxa expression even in mutated p53 cells, possibly through the regulation of E2F-1 [7]. Currently, and in contrast to the antiapoptotic gene *survivin *that is a direct target of Notch [15], no direct transcriptional regulation of Bcl-2 family genes by Notch has been reported. However, numerous regulators of Noxa expression have been described, among them p53, c-myc, and E2F-1 [reviewed in [33]], and further experiments are needed to investigate whether these, or additional factors, are involved in Noxa induction on Notch inhibition.

Importantly, Noxa specifically inhibits the survival activity of Mcl-1 and can also target it for proteosomal degradation [34]. This presumably occurs in GSIXII-treated cells, because we found that induced Noxa potently binds to Mcl-1. This event is a prerequisite for cell death induced by various stimuli (UV, cytokine deprivation, or treatment with anticancer agents) [reviewed in [33]]. Thus our work establishes that GSIXII, which triggers Noxa expression, functions as an indirect inhibitor of one key survival protein, Mcl-1. Because evasion of apoptosis has been recognized as one of the hallmarks of cancer, pharmacologic inhibition of antiapoptotic proteins is a potential strategy to restore apoptosis function in cancer cells. Several molecules, including ABT-737, have been designed to mimic the binding of BH3-only proteins to the hydrophobic groove of antiapoptotic proteins, blocking their activity. ABT-737 nevertheless binds preferentially to Bcl-2 and Bcl-xL but not to Mcl-1. Thus ABT-737 is ineffective in killing tumor cells expressing high levels of Mcl-1 compared with those of Bcl-2/Bcl-xL [35].

Breast cancer cells often express high levels of Bcl-2, Bcl-xL, and/or Mcl-1 [36]. Therefore, a rationale exists to use BH3 mimetics to circumvent apoptosis resistance in these cancers. We recently reported, in particular, that Mcl-1 participates in survival maintenance of breast cancer cells, at the very least in that of the HER2-amplified subtype [23]. Thus, combining Bcl-2/Bcl-xL and Mcl-1 inhibition by ABT-737 and GSIXII, respectively, should restore apoptosis sensitivity efficiently and affect survival maintenance, in breast cancer cells. Our results are consistent with this and clearly indicate that ABT-737 and GSIXII co-treatment led to synergistic apoptosis in breast tumors, suggesting the potential use of this combination to overcome cellular resistance. Interestingly, previous reports indicated that GSI treatment sensitized cancer cells to other chemotherapeutics drugs, such as oxaliplatin or 5-FU in colon cancer cells [16]. In the same line, the combination of BH3 mimetics with potent inducers of Noxa, such as vinblastin [37] or cisplatin [38], induced cancer cell sensitization to apoptosis.

Mammary stem cells, defined by indefinite self-replication that ensures tissue self-renewing by asymmetric cell division and generation of progenitor cells, have been isolated from both normal breast tissues and breast tumors [10]. This cell population exhibits an inherent capacity to form clonal mammospheres in suspension in *in vitro *assays, and in breast cancer stem cells, to initiate tumors in *in vivo *assays. Importantly, these cells show resistance to toxic agents [39]. Indeed, conventional chemotherapy often kills a majority of differentiated cancer cells but spares cancer stem cells, thus probably participating in cancer recurrence. We assessed this cellular compartment by using mammosphere-formation assay and showed that inhibition of Notch signaling, by using either GSIXII or SAHM1, successfully decreased mammosphere formation. This highlights the importance of the Notch pathway in mammary stem cell maintenance, as previously reported in breast cancer stem-like cells [40] or in normal mammary stem cells [10]. In addition, we found evidence that Noxa is involved in the effects of GSIXII, at least in part, because its knockdown significantly rescued mammosphere formation in GSIXII-treated cells. However, we cannot exclude the involvement of other mechanisms in the process, because this rescue was partial. Of particular interest, the simultaneous treatment with GSIXII and ABT-737 strongly impaired mammosphere formation. These results revealed that the Bcl-2 family of proteins might play an important role in maintaining the survival of breast cancer stem-like cells. Interestingly, this observation is supported by a recent report that shows that the co-silencing of Bcl-2, Bcl-xL, and Mcl-1 in breast cancer cell lines potently reduced mammosphere formation [41]. Practically, targeting breast cancer stem-like cells with BH3-mimetics should improve therapeutic outcomes.

Recent data suggest that Notch signaling is also important in the tumor microenvironment, as observed in myeloma [42] or head and neck squamous carcinoma cells [43]. The 3D short-term *ex vivo *model we developed, similar to the one described by van der Kuip and colleagues [44], allowed correct maintenance of intact breast tumoral tissues where cells remained viable and still proliferated because of sufficient diffusion of oxygen or nutrients. This assay preserves specific interactions between tumor cells and surrounding nontumoral tissue components and provides a powerful, rapid, and reproducible tool for studying the differential responses of individual tumors (and their various components) to specific drugs. Our results clearly demonstrate that GSI treatment is efficient in breast cancer cells embedded in their microenvironment.

We evaluated a series of 30 human primary breast tumors in the *ex vivo *assay and found evidence that 24 tumors exhibited high levels of caspase-3 activity on GSI treatment. Crucially, this method can be used to predict tumor sensitivity to drugs in a patient-specific manner and to help to identify patients who could benefit from the specific therapy. Moreover, combining GSIXII treatment with ABT-737 treatment led to a synergistic proapoptotic effect in six tumors tested. Among them, three were resistant to GSIXII, and four were resistant to ABT-737, each used as single agent. Thus, these results strongly argue for potent proapoptotic cooperation between GSIXII and ABT-737 in breast cancer cells maintained in their microenvironment.

## Conclusions

Altogether, our data provide strong evidence that γ-secretase inhibition triggers potent apoptosis in breast cancer cells. Moreover, the induction of Noxa expression played a major role in this process. Combining GSIXII treatment with ABT-737 strongly enhanced the apoptotic response in breast cancer cells, especially in tumors for which both molecules used as single agents led to a moderate proapoptotic effect. Thus, our data suggest that γ-secretase inhibition might offer a potent novel approach to treating breast cancers. Experimental treatments with Notch inhibitors in animal models were very promising [45]. However, they resulted in serious gastrointestinal side effects or immunosuppression [46]. A therapeutic window may exist if GSI could be given for short periods or in smaller doses. On the basis of our data, we propose that combining γ-secretase inhibition with Bcl-2/Bcl-xL targeting, might allow us to use concentrations of GSI under the side-effect limit in breast cancer therapy.

## Abbreviations

BH3 domain: Bcl-2-homology 3 domain; ER: estrogen receptor; GSI: γ-secretase inhibitor; HER2: epidermal growth factor receptor 2; HES: hematoxylin-eosin-saffron; IHC: immunohistochemical analysis; MFU: mammosphere-forming unit; NICD: Notch intracellular domain; PVDF: polyvinylidene fluoride; SDS-PAGE: sodium dodecylsulfate polyacrylamide gel electrophoresis; 2D or 3D: 2- or 3-dimensional.

## Competing interests

The authors declare that they have no competing interests.

## Authors' contributions

CS performed the majority of *in vitro *experiments and improved the *ex vivo *assay. DL conducted tumor histology analysis. SB participated in immunoblot experiments. MC helped to obtain human breast tumors. SBN performed mammosphere assays. PJ and SBN conceived the study and participated in the design of the project and the preparation of the manuscript. All authors read and approved the final manuscript.

## Supplementary Material

Additional file 1 **GSIXII treatment induced Annexin-V-positive staining in breast cancer cells**. Cells were incubated with 15 μ*M *GSIXII or with DMSO for 48 hours, and then assessed for Annexin-V expression with flow cytometry. Click here for file

Additional file 2 **Proteasome activity inhibition and apoptosis induction on GSIXII or bortezomib treatment did not correlate in breast cancer cell lines**. Proteolytic activity of 20S proteasome was quantified in breast cancer cell lines treated with GSIXII (15 μ*M*) or bortezomib (10 n*M*) with the fluorimetric substrate assay by using the substrate Suc-Leu-Leu-Val-Leu-AMC, according to the manufacturer's recommendation (Tebu Bio, Le Perray-en-Yvelines, France) **(A)**. Apo2.7-positive cells were evaluated on 15 μ*M *GSIXIII treatment or 10 n*M *bortezomib for 48 hours, as previously described **(B)**. Data are represented as percentage mean of inhibition compared with control (mock-treated) cells ± SEM; *n *= 3. Click here for file

Additional file 3 **BH3-only proteins expression in MDAMB231 cells after RNA interference**. Extinction of proteins expression was evaluated with immunoblot analysis after siRNA transfection in MDAMB231 for Bim and Puma **(A) **and Noxa on GSIXII treatment or not for 48 hours **(B)**. Click here for file

Additional file 4 **GSIXII treatment did not induce other Puma and Bim BH3-only proteins**. Expression of Noxa, Puma, and Bim proteins was assessed with immunoblot after 48 hours of treatment of GSIXII in MDAMB231 cells. *Nonspecific band. Click here for file

Additional file 5 **DAPT synergized with ABT-737 to trigger apoptosis in breast cancer cells**. MDAMB231 cells were incubated for 48 hours with 10 μ*M *DAPT (Sigma, Saint-Quentin Fallavier, France) in combination or not with ABT-737, 1 μ*M*. Then apoptosis was evaluated with Apo2.7 immunostaining and flow-cytometry analysis. Represented data are the means of positive cells ± SEM, from three independent experiments. Click here for file

Additional file 6 **Noxa co-immunoprecipitated mainly with Mcl-1 after GSIXIII treatment**. Cells were treated for 48 hours with GSIXII and QVD-OPH (to avoid cell death and obtain sufficient protein material in treated cells) before lysis in CHAPS buffer. Whole lysates were incubated overnight with the capture antibody (Mcl-1 S19 clone; Santa Cruz (Santa Cruz, USA), and then immunocomplexes were captured by using protein G-magnetic beads according to manufacturer's instructions (Millipore, Molsheim, France), in GSIXII-treated or untreated indicated cells. The immunoprecipitates were analyzed for the presence of Mcl-1 and Noxa proteins with immunoblotting. Click here for file

Additional file 7 **Correlation between HES and active caspase-3 IHC on GSIXII-treated tumors**. Each specimen was scored for active caspase-3 IHC and HES staining, allowing cell-morphology analysis. Active caspase-3 was scored as percentage of positive tumor cells. HES score was established in three groups, depending on the percentage of cells with altered morphology: group 1 (< 25%), group 2 (25% to 50%), and group 3 (> 50%). Click here for file

## References

[B1] Ranganathan P Weaver KL Capobianco AJ Notch signalling in solid tumours: a little bit of everything but not all the time Nature Rev Cancer 2011 11 338 351 10.1038/nrc3035 21508972

[B2] Ellisen LW Bird J West DC Soreng AL Reynolds TC Smith SD Sklar J TAN-1, the human homolog of the Drosophila notch gene, is broken by chromosomal translocations in T lymphoblastic neoplasms Cell 1991 66 649 661 10.1016/0092-8674(91)90111-B 1831692

[B3] Weng AP Ferrando AA Lee W Morris JP Silverman LB Sanchez-Irizarry C Blacklow SC Look AT Aster JC Activating mutations of NOTCH1 in human T cell acute lymphoblastic leukemia Science 2004 306 269 271 10.1126/science.1102160 15472075

[B4] Gallahan D Jhappan C Robinson G Hennighausen L Sharp R Kordon E Callahan R Merlino G Smith GH Expression of a truncated Int3 gene in developing secretory mammary epithelium specifically retards lobular differentiation resulting in tumorigenesis Cancer Res 1996 56 1775 1785 8620493

[B5] Reedijk M Odorcic S Chang L Zhang H Miller N McCready DR Lockwood G Egan SE High-level coexpression of JAG1 and NOTCH1 is observed in human breast cancer and is associated with poor overall survival Cancer Res 2005 65 8530 8537 10.1158/0008-5472.CAN-05-1069 16166334

[B6] Pece S Serresi M Santolini E Capra M Hulleman E Galimberti V Zurrida S Maisonneuve P Viale G Di Fiore PP Loss of negative regulation by Numb over Notch is relevant to human breast carcinogenesis J Cell Biol 2004 167 215 221 10.1083/jcb.200406140 15492044 PMC2172557

[B7] Rizzo P Miao H D'Souza G Osipo C Song LL Yun J Zhao H Mascarenhas J Wyatt D Antico G Hao L Yao K Rajan P Hicks C Siziopikou K Selvaggi S Bashir A Bhandari D Marchese A Lendahl U Qin JZ Tonetti DA Albain K Nickoloff BJ Miele L Cross-talk between notch and the estrogen receptor in breast cancer suggests novel therapeutic approaches Cancer Res 2008 68 5226 5235 10.1158/0008-5472.CAN-07-5744 18593923 PMC4445363

[B8] Hao L Rizzo P Osipo C Pannuti A Wyatt D Cheung LW Sonenshein G Osborne BA Miele L Notch-1 activates estrogen receptor-alpha-dependent transcription via IKKalpha in breast cancer cells Oncogene 2010 29 201 213 10.1038/onc.2009.323 19838210 PMC4976641

[B9] Osipo C Patel P Rizzo P Clementz AG Hao L Golde TE Miele L ErbB-2 inhibition activates Notch-1 and sensitizes breast cancer cells to a gamma-secretase inhibitor Oncogene 2008 27 5019 5032 10.1038/onc.2008.149 18469855

[B10] Dontu G Jackson KW McNicholas E Kawamura MJ Abdallah WM Wicha MS Role of Notch signaling in cell-fate determination of human mammary stem/progenitor cells Breast Cancer Res 2004 6 R605 R615 10.1186/bcr920 15535842 PMC1064073

[B11] Tanos T Brisken C What signals operate in the mammary niche? Breast Dis 2008 29 69 82 19029626 10.3233/bd-2008-29108

[B12] Kopan R Ilagan MX The canonical Notch signaling pathway: unfolding the activation mechanism Cell 2009 137 216 233 10.1016/j.cell.2009.03.045 19379690 PMC2827930

[B13] Meurette O Stylianou S Rock R Collu GM Gilmore AP Brennan K Notch activation induces Akt signaling via an autocrine loop to prevent apoptosis in breast epithelial cells Cancer Res 2009 69 5015 5022 19491273 10.1158/0008-5472.CAN-08-3478

[B14] Rasul S Balasubramanian R Filipović A Slade MJ Yagüe E Coombes RC Inhibition of gamma-secretase induces G2/M arrest and triggers apoptosis in breast cancer cells Br J Cancer 2009 100 1879 1888 10.1038/sj.bjc.6605034 19513078 PMC2714234

[B15] Lee CW Raskett CM Prudovsky I Altieri DC Molecular dependence of estrogen receptor-negative breast cancer on a notch-survivin signaling axis Cancer Res 2008 68 5273 5281 10.1158/0008-5472.CAN-07-6673 18593928 PMC2652573

[B16] Meng RD Shelton CC Li YM Qin LX Notterman D Paty PB Schwartz GK gamma-Secretase inhibitors abrogate oxaliplatin-induced activation of the Notch-1 signaling pathway in colon cancer cells resulting in enhanced chemosensitivity Cancer Res 2009 69 573 582 10.1158/0008-5472.CAN-08-2088 19147571 PMC3242515

[B17] Stylianou S Clarke RB Brennan K Aberrant activation of Notch signaling in human breast cancer Cancer Res 2006 66 1517 1525 10.1158/0008-5472.CAN-05-3054 16452208

[B18] Harrison H Farnie G Brennan KR Clarke RB Breast cancer stem cells: something out of notching? Cancer Res 2010 70 8973 8976 10.1158/0008-5472.CAN-10-1559 21045140

[B19] Hanahan D Weinberg RA The hallmarks of cancer Cell 2000 100 57 70 10.1016/S0092-8674(00)81683-9 10647931

[B20] Du H Wolf J Schafer B Moldoveanu T Chipuk JE Kuwana T BH3 domains other than Bim and Bid can directly activate Bax/Bak J Biol Chem 2011 286 491 501 10.1074/jbc.M110.167148 21041309 PMC3013008

[B21] Letai AG Diagnosing and exploiting cancer's addiction to blocks in apoptosis Nat Rev Cancer 2008 8 121 132 10.1038/nrc2297 18202696

[B22] Oltersdorf T Elmore SW Shoemaker AR Armstrong RC Augeri DJ Belli BA Bruncko M Deckwerth TL Dinges J Hajduk PJ Joseph MK Kitada S Korsmeyer SJ Kunzer AR Letai A Li C Mitten MJ Nettesheim DG Ng S Nimmer PM O'Connor JM Oleksijew A Petros AM Reed JC Shen W Tahir SK Thompson CB Tomaselli KJ Wang B Wendt MD An inhibitor of Bcl-2 family proteins induces regression of solid tumours Nature 2005 435 677 681 10.1038/nature03579 15902208

[B23] Campone M Noël B Grau M Guillemin Y Gautier F Gouraud W Charbonnel C Campion L Jézéquel P Braun F Barré B Coqueret O Barillé-Nion S Juin P c-Myc dependent expression of proapoptotic Bim renders HER2-overexpressing breast cancer cell dependent on antiapoptotic Mcl-1 Mol Cancer 2011 10 110 10.1186/1476-4598-10-110 21899728 PMC3175201

[B24] Ostasiewicz P Zielinska DF Mann M Wiśniewski JR Proteome, phosphoproteome, and N-glycoproteome are quantitatively preserved in formalin-fixed paraffin-embedded tissue and analyzable by high-resolution mass spectrometry J Proteome Res 2010 9 3688 3700 10.1021/pr100234w 20469934

[B25] de Carné Trécesson S Guillemin Y Bélanger A Bernard AC Preisser L Ravon E Gamelin E Juin P Barré B Coqueret O Escape from p21-mediated oncogene-induced senescence leads to cell dedifferentiation and dependence on anti-apoptotic Bcl-xL and MCL1 proteins J Biol Chem 2011 286 12825 12838 10.1074/jbc.M110.186437 21292770 PMC3075630

[B26] Hsieh JJ Henkel T Salmon P Robey E Peterson MG Hayward SD Truncated mammalian Notch1 activates CBF1/RBPJk-repressed genes by a mechanism resembling that of Epstein-Barr virus EBNA2 Mol Cell Biol 1996 16 952 959 8622698 10.1128/mcb.16.3.952 PMC231077

[B27] Yu X Alder JK Chun JH Friedman AD Heimfeld S Cheng L Civin CI HES1 inhibits cycling of hematopoietic progenitor cells via DNA binding Stem Cells 2006 24 876 888 10.1634/stemcells.2005-0598 16513761

[B28] Moellering RE Cornejo M Davis TN Del Bianco C Aster JC Blacklow SC Kung AL Gilliland DG Verdine GL Bradner JE Direct inhibition of the NOTCH transcription factor complex Nature 2009 462 182 188 10.1038/nature08543 19907488 PMC2951323

[B29] Han J Ma I Hendzel MJ Allalunis-Turner J The cytotoxicity of gamma-secretase inhibitor I to breast cancer cells is mediated by proteasome inhibition, not by gamma-secretase inhibition Breast Cancer Res 2009 11 R57 10.1186/bcr2347 19660128 PMC2750119

[B30] Farnie G Clarke RB Spence K Pinnock N Brennan K Anderson NG Bundred NJ Novel cell culture technique for primary ductal carcinoma in situ: role of Notch and epidermal growth factor receptor signaling pathways J Natl Cancer Inst 2007 99 616 627 10.1093/jnci/djk133 17440163

[B31] Harrison H Farnie G Howell SJ Rock RE Stylianou S Brennan KR Bundred NJ Clarke RB Regulation of breast cancer stem cell activity by signaling through the Notch4 receptor Cancer Res 2010 70 709 718 10.1158/0008-5472.CAN-09-1681 20068161 PMC3442245

[B32] Qin JZ Stennett L Bacon P Bodner B Hendrix MJ Seftor RE Seftor EA Margaryan NV Pollock PM Curtis A Trent JM Bennett F Miele L Nickoloff BJ p53-independent NOXA induction overcomes apoptotic resistance of malignant melanomas Mol Cancer Ther 2004 8 895 902 15299072

[B33] Ploner C Kofler R Villunger A Noxa: at the tip of the balance between life and death Oncogene 2008 27 Suppl 1 S84 S92 19641509 10.1038/onc.2009.46 PMC3272398

[B34] Czabotar PE Lee EF van Delft MF Day CL Smith BJ Huang DC Fairlie WD Hinds MG Colman PM Structural insights into the degradation of Mcl-1 induced by BH3 domains Proc Natl Acad Sci USA 2007 104 6217 6222 10.1073/pnas.0701297104 17389404 PMC1851040

[B35] Konopleva M Contractor R Tsao T Samudio I Ruvolo PP Kitada S Deng X Zhai D Shi YX Sneed T Verhaegen M Soengas M Ruvolo VR McQueen T Schober WD Watt JC Jiffar T Ling X Marini FC Harris D Dietrich M Estrov Z McCubrey J May WS Reed JC Andreeff M Mechanisms of apoptosis sensitivity and resistance to the BH3 mimetic ABT-737 in acute myeloid leukemia Cancer Cell 2006 10 375 388 10.1016/j.ccr.2006.10.006 17097560

[B36] Oakes SR Vaillant F Lim E Lee L Breslin K Feleppa F Deb S Ritchie ME Takano E Ward T Fox SB Generali D Smyth GK Strasser A Huang DC Visvader JE Lindeman GJ Sensitization of BCL-2-expressing breast tumors to chemotherapy by the BH3 mimetic ABT-737 Proc Natl Acad Sci USA 2012 109 2766 2771 10.1073/pnas.1104778108 21768359 PMC3286989

[B37] Albershardt TC Salerni BL Soderquist RS Bates DJ Pletnev AA Kisselev AF Eastman A Multiple BH3 mimetics antagonize antiapoptotic MCL1 protein by inducing the endoplasmic reticulum stress response and up-regulating BH3-only protein NOXA J Biol Chem 2011 286 24882 24895 10.1074/jbc.M111.255828 21628457 PMC3137063

[B38] Bray K Chen HY Karp CM May M Ganesan S Karantza-Wadsworth V DiPaola RS White E Bcl-2 modulation to activate apoptosis in prostate cancer Mol Cancer Res 2009 7 1487 1496 10.1158/1541-7786.MCR-09-0166 19737977 PMC2855683

[B39] Gupta PB Onder TT Jiang G Tao K Kuperwasser C Weinberg RA Lander ES Identification of selective inhibitors of cancer stem cells by high-throughput screening Cell 2009 138 645 659 10.1016/j.cell.2009.06.034 19682730 PMC4892125

[B40] Grudzien P Lo S Albain KS Robinson P Rajan P Strack PR Golde TE Miele L Foreman KE Inhibition of Notch signaling reduces the stem-like population of breast cancer cells and prevents mammosphere formation Anticancer Res 2010 30 3853 3867 21036696

[B41] Lang JY Hsu JL Meric-Bernstam F Chang CJ Wang Q Bao Y Yamaguchi H Xie X Woodward WA Yu D Hortobagyi GN Hung MC BikDD eliminates breast cancer initiating cells and synergizes with lapatinib for breast cancer treatment Cancer Cell 2011 20 341 356 10.1016/j.ccr.2011.07.017 21907925 PMC3172580

[B42] Houde C Li Y Song L Barton K Zhang Q Godwin J Nand S Toor A Alkan S Smadja NV Avet-Loiseau H Lima CS Miele L Coignet LJ Overexpression of the NOTCH ligand JAG2 in malignant plasma cells from multiple myeloma patients and cell lines Blood 2004 104 3697 3704 10.1182/blood-2003-12-4114 15292061

[B43] Zeng Q Li S Chepeha DB Giordano TJ Li J Zhang H Polverini PJ Nor J Kitajewski J Wang CY Crosstalk between tumor and endothelial cells promotes tumor angiogenesis by MAPK activation of Notch signaling Cancer Cell 2005 8 13 23 10.1016/j.ccr.2005.06.004 16023595

[B44] van der Kuip H Mürdter TE Sonnenberg M McClellan M Gutzeit S Gerteis A Simon W Fritz P Aulitzky WE Short term culture of breast cancer tissues to study the activity of the anticancer drug taxol in an intact tumor environment BMC Cancer 2006 6 86 97 10.1186/1471-2407-6-86 16603054 PMC1456977

[B45] Cullion K Draheim KM Hermance N Tammam J Sharma VM Ware C Nikov G Krishnamoorthy V Majumder PK Kelliher MA Targeting the Notch1 and mTOR pathways in a mouse T-ALL model Blood 2009 113 6172 6181 10.1182/blood-2008-02-136762 19246562 PMC2699237

[B46] van Es JH van Gijn ME Riccio O van den Born M Vooijs M Begthel H Cozijnsen M Robine S Winton DJ Radtke F Clevers H Notch/gamma-secretase inhibition turns proliferative cells in intestinal crypts and adenomas into goblet cells Nature 2005 435 959 963 10.1038/nature03659 15959515

