# Supplementary figures and images for: γ-Secretase inhibition promotes cell death, Noxa upregulation, and sensitization to BH3 mimetic ABT-737 in human breast cancer cells
Source: Breast Cancer Res. 2012 Jun 15;14(3):R96. doi: 10.1186/bcr3214 (PMC3446359; doi:10.1186/bcr3214)

Additional file 1


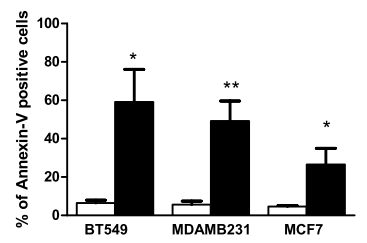

Supplement: Additional file 1 — GSIXII treatment induced Annexin-V-positive staining in breast cancer cells. Cells were incubated with 15 μM GSIXII or with DMSO for 48 hours, and then assessed for Annexin-V expression with flow cytometry. [file bcr3214-S1.DOCX]

Additional file 2

A


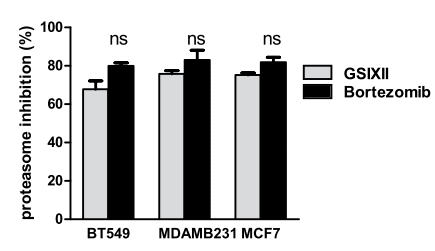


B


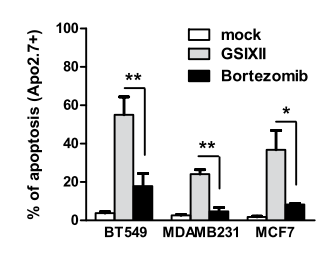

Supplement: Additional file 2 — Proteasome activity inhibition and apoptosis induction on GSIXII or bortezomib treatment did not correlate in breast cancer cell lines. Proteolytic activity of 20S proteasome was quantified in breast cancer cell lines treated with GSIXII (15 μM) or bortezomib (10 nM) with the fluorimetric substrate assay by using the substrate Suc-Leu-Leu-Val-Leu-AMC, according to the manufacturer's recommendation (Tebu Bio, Le Perray-en-Yvelines, France) (A). Apo2.7-positive cells were evaluated on 15 μM GSIXIII treatment or 10 nM bortezomib for 48 hours, as previously described (B). Data are represented as percentage mean of inhibition compared with control (mock-treated) cells ± SEM; n = 3. [file bcr3214-S2.DOCX]

Additional file 3

A


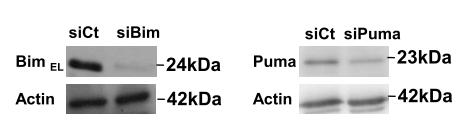


B


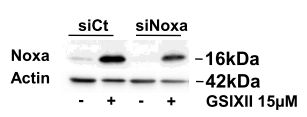

Supplement: Additional file 3 — BH3-only proteins expression in MDAMB231 cells after RNA interference. Extinction of proteins expression was evaluated with immunoblot analysis after siRNA transfection in MDAMB231 for Bim and Puma (A) and Noxa on GSIXII treatment or not for 48 hours (B). [file bcr3214-S3.DOCX]

Additional file 4


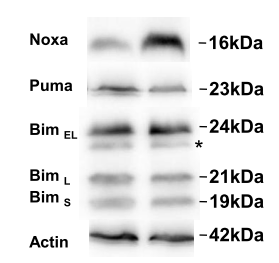

Supplement: Additional file 4 — GSIXII treatment did not induce other Puma and Bim BH3-only proteins. Expression of Noxa, Puma, and Bim proteins was assessed with immunoblot after 48 hours of treatment of GSIXII in MDAMB231 cells. *Nonspecific band. [file bcr3214-S4.DOCX]

Additional file 5


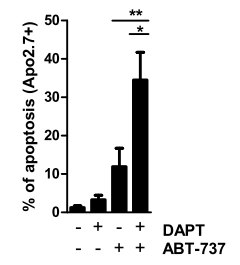

Supplement: Additional file 5 — DAPT synergized with ABT-737 to trigger apoptosis in breast cancer cells. MDAMB231 cells were incubated for 48 hours with 10 μM DAPT (Sigma, Saint-Quentin Fallavier, France) in combination or not with ABT-737, 1 μM. Then apoptosis was evaluated with Apo2.7 immunostaining and flow-cytometry analysis. Represented data are the means of positive cells ± SEM, from three independent experiments. [file bcr3214-S5.DOCX]

Additional file 6


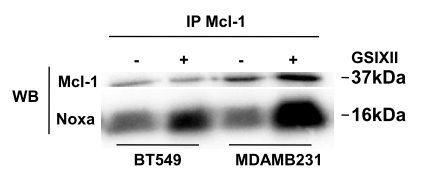

Supplement: Additional file 6 — Noxa co-immunoprecipitated mainly with Mcl-1 after GSIXIII treatment. Cells were treated for 48 hours with GSIXII and QVD-OPH (to avoid cell death and obtain sufficient protein material in treated cells) before lysis in CHAPS buffer. Whole lysates were incubated overnight with the capture antibody (Mcl-1 S19 clone; Santa Cruz (Santa Cruz, USA), and then immunocomplexes were captured by using protein G-magnetic beads according to manufacturer's instructions (Millipore, Molsheim, France), in GSIXII-treated or untreated indicated cells. The immunoprecipitates were analyzed for the presence of Mcl-1 and Noxa proteins with immunoblotting. [file bcr3214-S6.DOCX]

Additional file 7


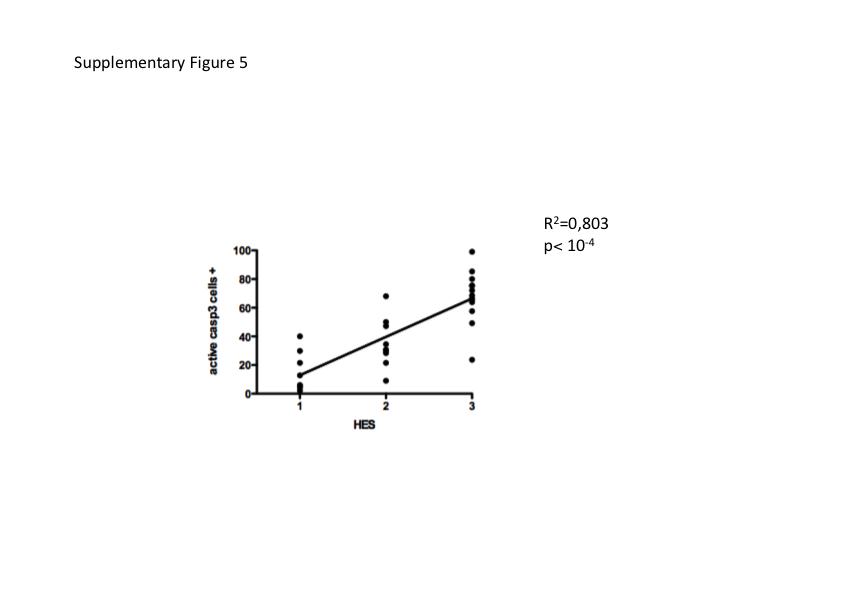

Supplement: Additional file 7 — Correlation between HES and active caspase-3 IHC on GSIXII-treated tumors. Each specimen was scored for active caspase-3 IHC and HES staining, allowing cell-morphology analysis. Active caspase-3 was scored as percentage of positive tumor cells. HES score was established in three groups, depending on the percentage of cells with altered morphology: group 1 (< 25%), group 2 (25% to 50%), and group 3 (> 50%). [file bcr3214-S7.DOCX]
